# Normal expression of DNA repair proteins, hMre11, Rad50 and Rad51 but protracted formation of Rad50 containing foci in X-irradiated skin fibroblasts from radiosensitive cancer patients

**DOI:** 10.1038/sj.bjc.6601878

**Published:** 2004-05-11

**Authors:** C Djuzenova, B Mühl, R Schakowski, U Oppitz, M Flentje

**Affiliations:** 1 Klinik für Strahlentherapie der Universität Würzburg, Josef-Schneider-Strasse 11, D-97080 Würzburg, Germany

**Keywords:** colony-forming assay, foci, radiosensitivity, Western blot, hMre11, Rad50, Rad51

## Abstract

About 5% of oncology patients treated by radiation therapy develop acute or late radiotoxic effects whose molecular mechanisms remain poorly understood. In this study, we evaluated the potential role of DNA repair proteins in the hypersensitivity of cancer patients to radiation therapy. The expression levels and focal nuclear distribution of DNA repair proteins, hMre11, Rad50 and Rad51 were investigated in skin fibroblasts strains derived from cancer patients with adverse early skin reaction to radiotherapy using Western blot and foci immunofluorescence techniques, respectively. Cells from cancer patients with normal reaction to radiotherapy as well as cells from apparently healthy subjects served as controls. Cellular radiosensitivity after *in vitro* irradiation was assessed by the clonogenic survival assay. The clonogenic survival assay and Western blot analysis of the DNA repair proteins did not reveal any abnormalities in cellular radiosensitivity *in vitro* and in protein expression levels or their migration patterns in the fibroblasts derived from cancer patients with hypersensitive reaction to radiotherapy. In contrast, *in vitro* irradiated cells from radiosensitive patients exhibited a significantly higher number of nuclei with focally concentrated Rad50 protein than in both control groups. The observed alteration of the distribution of radiation-induced Rad50 foci in cells derived from cancer patients with acute side reactions to radiotherapy might contribute to their radiation therapy outcome. These data suggest the usefulness of the Rad50 foci analysis for predicting clinical response of cancer patients to radiotherapy.

The reaction of healthy tissue to ionising radiation (IR) is one of the major factors determining the radiotherapy (RT) schedule and outcome. During or after RT, up to 5% of cancer patients (Norman *et al*, 1988) develop either acute radiotoxic responses, such as erythema and desquamation of the exposed skin as well as mucosa, or late adverse reactions, such as fibrosis and telangiectasias. So the doses of radiation used clinically have been determined on the basis of an ‘acceptable’ level of adverse skin reactions in 5% of patients.

There is a large body of evidence in the literature suggesting a genetic basis for the predisposition to side effects of RT in healthy tissue (McKay and Peters, 1997). Thus, dysfunction of genes and their protein products involved in the recognition and processing of the cellular radiation damage could be a possible molecular basis underlying the adverse radiotoxic reactions in healthy tissue. Despite many clinical and experimental efforts, including screening of the candidate radiosensitivity genes for mutations (Appleby *et al*, 1997; Oppitz *et al*, 1999), screening for abnormalities in key candidate proteins (Carlomagno *et al*, 2000; Leong *et al*, 2003), functional genomics using DNA-microarrays (Kitahara *et al*, 2002), induction of DNA double-strand breaks (DSBs) (El-Awady *et al*, 2003), the molecular mechanisms of clinical radiosensitivity remain poorly understood.

DNA double-strand breaks are biologically the most important lesions produced by IR and other exogenous agents, and they are the major threats to the genomic integrity of cells (Kanaar *et al*, 1998). If insufficiently repaired or misrepaired, DSBs may lead to chromosome breaks, deletions and translocations (Dasika *et al*, 1999). There are at least two distinct pathways for DSBs repair in eucaryotic cells. These are the error-prone nonhomologous end-joining (NHEJ) and error-free homologous recombination (HR) (Dasika *et al*, 1999). Until recently, the NHEJ mechanism, which includes the Ku heterodimer (Ku70 and Ku80), XRCC4 and DNA ligase IV (for a review, see Jeggo, 1998), was thought to be the primary mechanism in mammalian cells for repairing DSBs (Jeggo, 1998; Thompson and Schild, 2001). However, accumulating experimental evidence indicates that the HR pathway is equally important (Liang *et al*, 1998; Takata *et al*, 1998). The relative contribution of NHEJ and HR varies during cell differentiation and is also dependent on the cell cycle stage. The NHEJ pathway plays a dominant role in repairing the *γ*-radiation-induced DSBs during G1 and early S phase, while HR preferentially operates in late S and G2 phase (Takata *et al*, 1998). The HR mechanism involves proteins of the Rad52 epistasis group genes (Rad50, RAD51, Rad52, Rad54, Rad55, Rad57, Rad59, Mre11 and Xrs2) and plays a crucial role in DSBs repair in higher eucaryotic cells (reviewed in Thompson and Schild, 2001). Rad51 is the major strand-transfer protein in eucaryotic cells and found to interact with many proteins, including c-Ab1, BRCA2, BLM, XRCC3, etc.

An intact Rad50–Mre11–NBS1 complex has been found to be essential for the normal DSBs repair (Petrini, 1999). Moreover, this complex participates in both DSBs repair pathways, even though they are mechanistically different. Recently, mutations in the *Mre11* gene have been identified in four patients from two families with an ‘ataxia telangiectasia-like disorder’ (ATLD) (Stewart *et al*, 1999). Although the cellular ATLD phenotype is similar to that of real ataxia telangiectasia cells, the ATLD mutant cells show lower radiation sensitivity. Disruption of the mammalian *Rad50* gene results in embryonic cell lethality and increased sensitivity to IR of explanted blastocytes (Luo *et al*, 1999). Thus, all embryo outgrowths containing mutation in mRad50 had lost their inner cell masses after 2 Gy of *γ*-irradiation (Luo *et al*, 1999).

Prediction of the radiation sensitivity based upon the expression of Rad51 protein is apparently not straightforward (Vispé *et al*, 1998; Yanagisawa *et al*, 1998; Yanez and Porter, 1999; Kim *et al*, 2001). Some authors have reported that an overexpression of this protein correlates with the increased cellular resistance against radiation (Vispé *et al*, 1998; Yanagisawa *et al*, 1998; Yanez and Porter, 1999). In contrast, other workers (e.g. Kim *et al*, 2001) have found that overexpression of human Rad51 in CHO cells reduces the repair of DSBs by homologous recombination.

In order to elucidate the molecular mechanisms underlying the increased clinical radiosensitivity, the DSBs repair proteins were analysed in the present study in fibroblast lines derived from patients with different reactions to RT. Owing to their involvement in both known DSBs repair pathways (NHEJ and HR), proteins hMre11 and Rad50 were selected as the promising molecular markers for screening abnormalities in the cellular radiation response. In addition, we choose Rad51 protein which is involved in HR. Using the Western blot and foci immunofluorescence techniques, the expression levels, electrophoretic migration patterns and subnuclear distributions of the three proteins were analysed in cells from cancer patients with increased and normal clinical radiosensitivity, as well as from healthy donors.

## MATERIAL AND METHODS

### Materials

Cell culture media, fetal bovine serum (FBS) and most chemicals were obtained from Sigma (Deisenhofen, Germany). If otherwise not stated, Falcon plasticware (Becton Dickinson Labware, Franklin Lakes, NJ, USA) was used in cell culture experiments.

### Subjects

The assay was performed on fibroblast strains that were initiated from skin biopsies of breast cancer patients with normal (grade 0–1 according RTOG score) and acute (grade 2–4 RTOG, see Table 1

**Table 1:** Cloning efficiencies, radiosensitivity parameters of irradiated *in vitro* skin fibroblasts and grade of early skin reactions to radiotherapy

**Strain**	**Clinical description**	**Plating efficiency (%)**	***α* (Gy^−1^)**	***β* (Gy^−2^)**	**SF2**	**RTOG, skin, early**^a^
1BR3	Normal	14.3±0.8	0.17±0.1	0.06±0.06	0.56	—
HFIB1	Normal	36.8±3.6	0.14±0.1	0.08±0.02	0.55	—
HSF1	Normal	12.3±5.1	0.27±0.08	0.05±0.02	0.48	—
F7	Normal	10.7±2.1	0.27±0.06	0.06±0.02	0.46	—
HFIB2	Normal	29.8±6.8	0.41±0.03	0.05±0.005	0.37	—
						
87HF	Breast cancer	7.4±1.0	0.37±0.04	0.03±0.001	0.42	1
88HF	Breast cancer	21.5±0.8	0.51±0.05	0.05±0.01	0.30	1–2
89HF	Breast cancer	22.0±0.3	0.45±0.04	0.03±0.01	0.36	1
94HF	Breast cancer	12.9±1.5	0.34±0.03	0.05±0.01	0.42	1
98HF	Breast cancer	20.1±3.1	0.63±0.06	0.02±0.012	0.27	1
121HF	Breast cancer	12.8±2.5	0.55±0.22	0.08±0.08	0.24	1
						
HS5	Breast cancer	16.0±3.0	0.58±0.05	0.05±0.02	0.26	2–3
HS6	Breast cancer	10.0±1.0	0.44±0.04	0.03±0.01	0.38	3
HS8	Breast cancer	11.5±2.1	0.35±0.02	0.06±0.006	0.40	3–4
HS13	Breast cancer	24.8±1.7	0.38±0.02	0.05±0.005	0.39	3
HS14	Breast cancer	11.8±1.9	0.43±0.02	0.04±0.004	0.36	3
HS15	Breast cancer	17.0±1.6	0.36±0.01	0.04±0.003	0.41	3
144	Breast cancer	17.2±1.8	0.71±0.04	0.05±0.01	0.20	2–3
148	Breast cancer	15.5±1.6	0.75±0.19	0.03±0.03	0.20	2

Mean (±s.e.) from at least two independent experiments.

a Early skin reaction according RTOG score (Cox *et al*, 1995). RTOG grade: 1 – follicular, faint or dull erythema, dry desquamation; 2 – tender or bright erythema, patchy moist desquamation, moderate edema; 3 – confluent, moist desquamation, pitting edema; 4 – ulceration, haemorrhage, necrosis.

) reaction of their skin to radiotherapy (see Table 1). The study was approved by the University of Würzburg Ethics Committee and all patients gave informed consent. Skin punch biopsies were obtained under local anaesthesia from the inner of the upper arm with a 4 mm punch-needle. The biopsy specimens were disaggregated mechanically and primary fibroblast cultures were initiated by an outgrowth technique in which skin samples were incubated in complete growth media (see below) in a humidified atmosphere with 5% CO_2_ in air at 37°C. The outgrowth lasted from 4 to 10 days and the cells were collected until they reach subconfluence after a mean period of 24 days (range 20–28 days). Skin fibroblast cultures derived from apparently healthy donors served as controls. These were: HSF1 and F7 (kindly gifted by Professor E Dikomey, University Hospital Hamburg-Eppendorf, Germany), 1BR3 (kindly gifted by Dr P Jeggo, Medical Research Council, Cell Mutation Unit, University of Sussex, Brighton, UK), HFIB1 and HFIB2 fibroblast lines were purchased from Cell-Lining GmbH (Berlin, Germany). Cells at passage from 3 to 12 were used for experiments.

### Culture handling

Monolayer cultures of each strain were grown in Dulbecco's modified Eagle's medium (Sigma D-6046, Deisenhofen, Germany,) supplemented with 10% FBS (Sigma F-7524), glutamine (1 mM) and penicillin–streptomycin (100 U ml^−1^ and 100 *μ*g ml^−1^, respectively), hereafter denoted as complete growth medium (CGM). Cultures were routinely incubated at 37°C in a humidified atmosphere enriched with 9% CO_2_. The cells that reached confluence after 2–4 days of incubation were passaged by mild trypsinisation (0.05% trypsin/0.02% EDTA, Sigma T-3924).

### Antibodies

Primary antibodies used were: rabbit polyclonal anti-Rad51 (1 : 100, Oncogene, PC130) and anti-hMre11 (1 : 100, Oncogene, PC388) and mouse monoclonal anti-Rad50 (1 : 1000, Abcam, ab145). Secondary species-specific antibodies were either labelled with horseradish-peroxidase (1 : 2000, DAKO, Hamburg, Germany) or with Alexa Fluor 488 and 568 nm (1 : 200, Molecular Probes, Eugene, OR, USA, A-11001 and A-11011) for Western blot and foci immunofluorescence detection, respectively.

### X-ray irradiation

Irradiation was performed at room temperature using a 6 MV Siemens linear accelerator (Siemens, Concord, CA, USA) at a dose rate of 2 Gy min^−1^. After irradiation, cells were recovered in CGM for the indicated time until harvested.

### X-irradiation and colony survival

Cell survival curves were generated by a standard colony-formation assay as previously described (Djuzenova and Flentje, 2002) with minor modifications. Precooled fibroblasts irradiated by graded single doses (0–8 Gy) were seeded in Petri dishes and cultivated in CGM. Three replicates were carried out for each exposure point, and the experiments were repeated at least twice. After 2 weeks, the cells were fixed and stained with crystal violet. Colonies of at least 50 cells were scored as survivors. The mean survival data for each individual cell line were fitted to the linear quadratic (LQ) model:


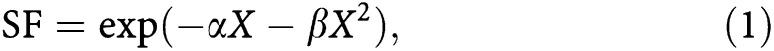


where, SF is the survival fraction, *X* is the irradiation dose, *α* and *β* are the fitted parameters.

### Western blot analysis

Nearly confluent nonirradiated and irradiated with 8 Gy fibroblasts were detached by trypsinisation, lyzed in RIPA buffer (10 mM tris-HCl, pH 7.4, 150 mM NaCl, 1% sodium deoxycholate, 1% Triton X-100) containing protease inhibitors (2 *μ*g ml^−1^ aprotinin, 2 *μ*g ml^−1^ leupeptin, 5 *μ*g ml^−1^ pepstatin, 1 mM phenylmethane sulphonyl fluoride) for 30 min on ice and subsequently centrifuged 10 min at 500 **g**. Samples equivalent to 20 *μ*g of protein were separated using either 4–12% (for hMre11 and Rad51) or 3–8% (for Rad50) sodium-dodecyl-sulphate–polyacrylamide precast gels (Invitrogen, Karlsruhe, Germany) and transferred to nitrocellulose membranes according to standard procedures. Equal loading and transfer were assessed by reprobing the blots with anti-*β*-actin antibody (Sigma A-5316) and Ponceau red (Sigma 19 976-1), respectively. *β*-Actin was used as an internal standard to account for variations in the amount of protein (usually 20 *μ*g) loaded in each lane. For hMre11, Rad51 or Rad50 detection, membranes were incubated with respective primary and species-specific peroxidase-labelled secondary antibodies according to standard procedures. Protein bands were detected using an enhanced chemiluminescence system (ECL, Amersham Pharmacia Biotech, Braunschweig, Germany). The levels of protein expression were quantified using the Kodak 1D Image analysing software (Scientific Imaging Systems, Eastman Kodak Company, Rochester, NY, USA) and normalised to the *β*-actin levels.

### hMre11, Rad51 and Rad50 foci formation

Fibroblasts were cultured on microscope glass slides for at least 24 h. Subconfluent cells were irradiated with 8 Gy on slides, fixed at various time points in ice-cold ethanol at −20°C, and permeabilised with Triton X-100 (1% solution in PBS) for 5 min. Slides were washed three times for 5 min in PBS and blocked in 4% FBS–PBS for 1 h at room temperature. Slides were incubated for 1 h at 37°C with anti-hMre11, -Rad50 or -Rad51 primary antibodies, followed by incubation with respective secondary antibodies conjugated with Alexa Fluor 488 nm or 568 nm. Slides were counterstained with 0.2 *μ*g ml^−1^ of DAPI (4′,6′-diamidino-2-phenylindole) in antifade solution (1.5% *N*-propyl-gallate, 60% glycerol in PBS) and examined using a Leica DMLB epifluorescence microscope coupled to a cooled CCD camera (ColorView 12, Olympus Biosystems, Hamburg, Germany). Camera control and image acquisition were carried out using an image analysis software (AnalySis, SoftImaging GmbH, Stuttgart, Germany). For each experimental point, at least 100 nuclei were examined and hMre11, Rad51 or Rad50 foci were scored by eye at a magnification of × 1000. Probes were then quantified by counting the number of foci-positive cells, and also by counting the number of foci per nucleus in the fraction of foci-positive cells. We used no threshold for foci number per nucleus.

### Statistics

Data are presented as mean (±s.d. or ±s.e.). The mean values were compared by the Student's *t*-test. The threshold of statistical significance was set at *P*<0.05. Statistics and fitting of experimental curves were performed with the program Origin (Microcal, Northampton, MA, USA).

## RESULTS

### Clonogenic survival

Figure 1

**Figure 1:**
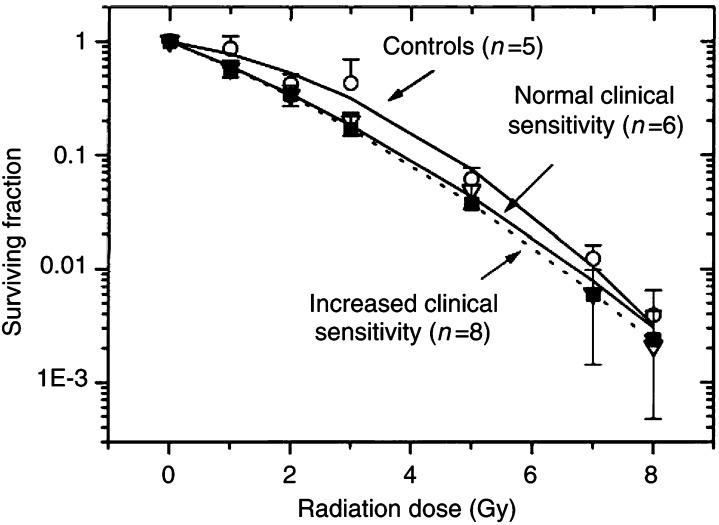
Survival curves of skin fibroblasts from control donors, cancer patients with normal reaction to RT and hypersensitive cancer patients as functions of the radiation dose. Irradiated cell were plated in CGM and incubated at 37°C in a humidified atmosphere with 9% CO_2_. After 2 weeks, the cells were fixed and stained using crystal violet. Colonies of at least 50 cells were scored as survivors. The data derived from two experiments for each strain were pooled together and fitted with a linear-quadratic equation (Eq. 1). Standard deviations are indicated by error bars.

shows the normalised survival responses of the fibroblast strains averaged through each group as functions of X-ray dose together with the best fits of the LQ model (Eq. 1). As judged by the correlation coefficients, ranging between 0.97 and 0.99, the LQ model (curves in Figure 1) provided reasonable approximations to the experimental data (symbols in Figure 1). Plating efficiencies of nonirradiated fibroblast strains, as well as the fitted parameters *α* and *β* obtained by the nonlinear regression analysis and the calculated surviving fractions at 2 Gy (SF2) for each individual cell strain are summarised in Table 1. Judging by the SF2 values, fibroblasts strains from clinically hypersensitive cancer patients with SF2=0.33±0.08 (mean±s.d., *n*=8) and from cancer patients with normal clinical sensitivity (SF2=0.34±0.07, *n*=6) were significantly more sensitive to X-irradiation than fibroblasts from healthy donors (SF2=0.48±0.07, *n*=5). However, in this rather small patient sample there was no difference in the SF2 values between fibroblasts of cancer patients with normal (SF2=0.34±0.07, *n*=6) and hypersensitive (SF2=0.33±0.08) reactions to RT.

In order to elucidate the cellular mechanisms underlying the different clinical reactions to RT, we further examined the expression levels and migration patterns of the three DNA DSBs repair proteins hMre11, RAd50 and Rad51 as well as their nuclear focal distribution before and after *in vitro* irradiation.

### Analysis of hMre11, Rad50 and Rad51 by Western blotting

Figure 2

**Figure 2:**
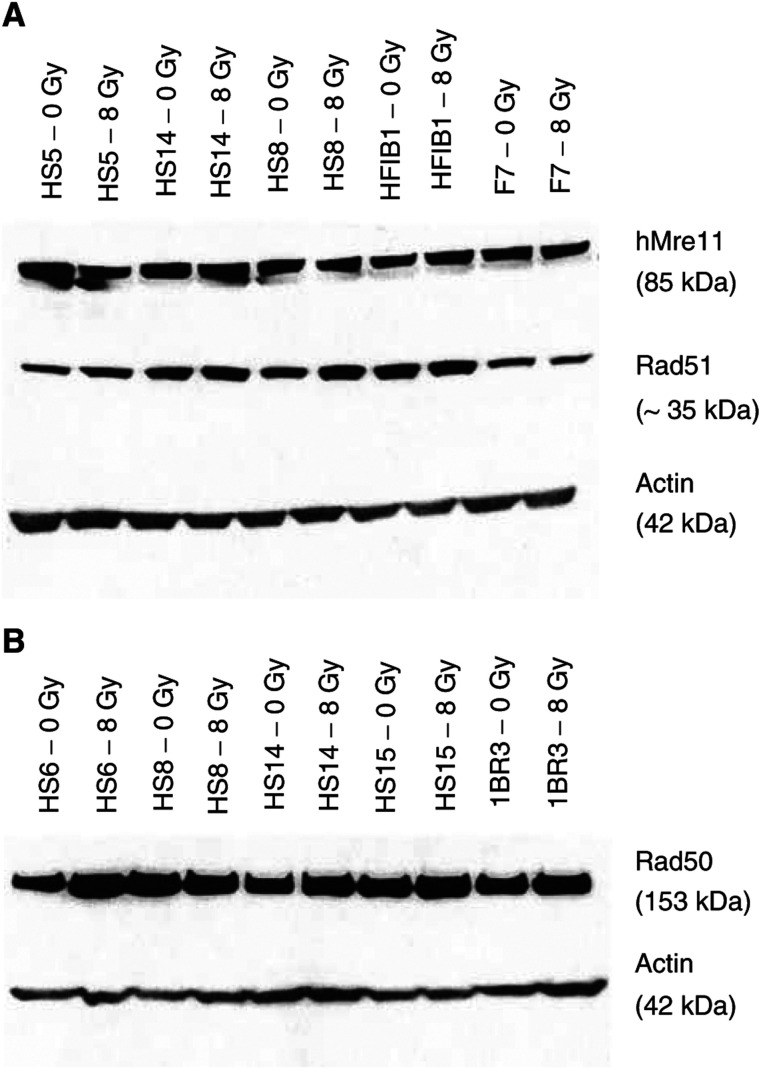
Western blot analysis of expression levels and migration patterns of hMre11 (**A**, top), Rad51 (**A**, middle) and Rad50 (**B**, top) proteins in nonirradiated and irradiated cells from control and radiosensitive cancer patients. *β*-Actin (bottom bands in **A** and **B**) was used as an internal standard to allow for differences in protein loading in each lane (20 *μ*g). There were no differences in expression of hMre11 and Rad51 between control (HFIB1 und F7) and (HS5, HS14, HS8) radiosensitive cell lines (**A**), as well as in expression of Rad50 (**B**) between control (1BR3) and radiosensitive (HS6, HS8, HS14, HS15) cell lines.

shows a typical Western blot analysis demonstrating the levels of protein expression in nonirradiated and irradiated with 8 Gy fibroblasts. It is obvious from Figure 2 that the expression levels of all tested proteins were mostly similar in the cells derived from healthy donors and cancer patients with increased reaction to RT. Such immunoblots were quantified and the amounts of hMre11, Rad51 and Rad50 were normalised to the respective levels of *β*-actin. Statistical analysis (Figure 3

**Figure 3:**
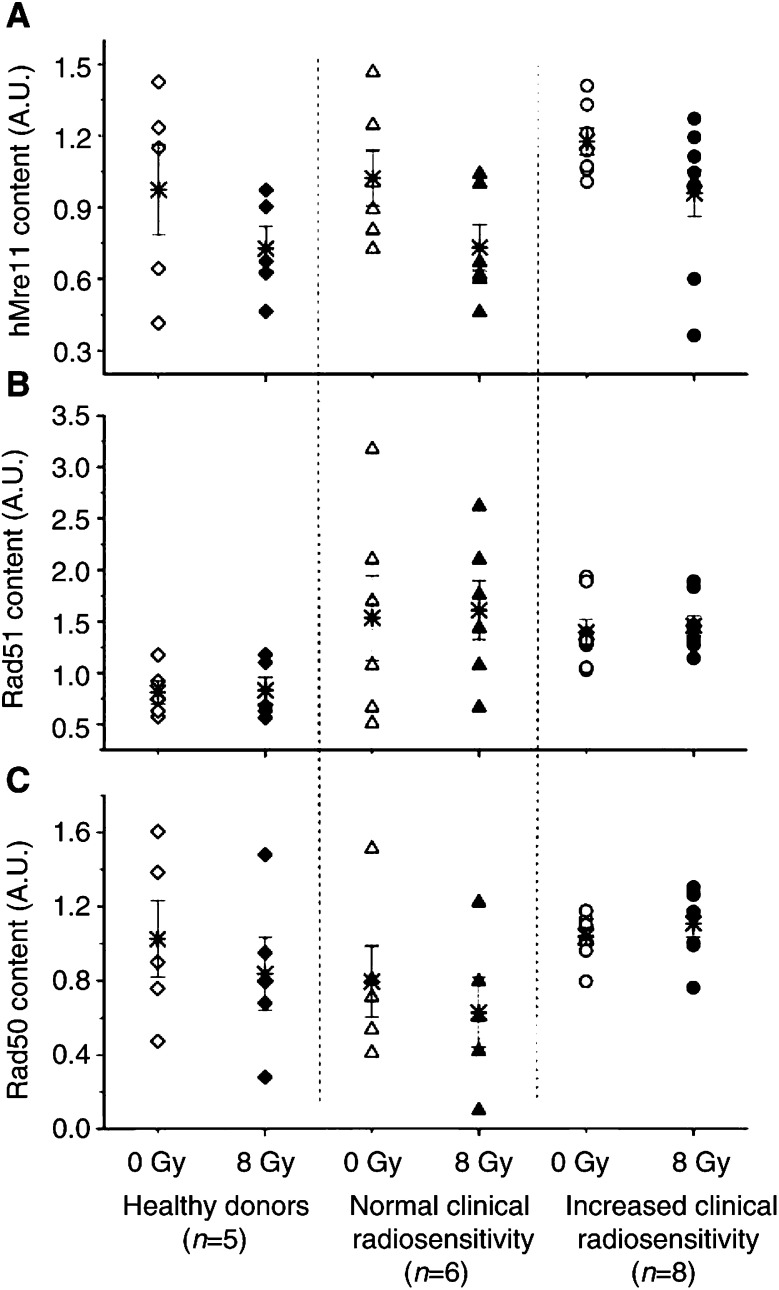
Expression of hMre11 (top), Rad51 (middle) and Rad50 (bottom) proteins normalised to *β*-actin before (open symbols) and 2 h after irradiation with 8 Gy (solid symbols) assessed by Western blot analysis of 14 cell lines with different sensitivities to RT and five control cell lines. Each symbol (except stars) represents the mean value obtained from at least four independent experiments on the cell strain from a given individual. Stars represent the mean value (±s.e.) averaged through each tested group. Western blotting did not reveal any differences in protein expression between the control group and cancer patients with normal and increased clinical sensitivity to RT.

) of the Western blot data through at least four (up to 12) independent experiments for each cell line and the group tested revealed that the mean levels of hMre11 protein were similar in healthy group (1.0±0.2) and in group of cancer patient with normal reaction to RT (1.0±0.1) before and after (0.7±0.1 in both groups of subjects) *in vitro* irradiation. Cells derived from hypersensitive cancer patients showed somewhat elevated levels of hMre11 before (1.2±0.1) and after (1.0±0.1) irradiation.

As seen from Figure 3B, the mean basal levels of Rad51 in cells derived from cancer patients with normal (1.5±0.4) and hypersensitive (1.4±0.1) reaction to RT was higher than that in cells derived from healthy donors (0.8±0.1). These differences, however, was not confirmed statistically. At 2 h postirradiation, the mean level of Rad51 in all cell lines remains similar to that before irradiation. The mean amounts of the Rad50 protein (Figure 3C, open symbols) were almost the same in nonirradiated cells derived from healthy subjects (1.0±0.2), cancer patients with normal (0.8±0.2) and increased clinical reaction (1.0±0.2). At 2 h postirradiation, the expression of Rad50 decreased by about 20% in cells from healthy donors and from cancer patients with normal reaction to RT. In contrast, the irradiated cells of hypersensitive cancer patients revealed a slightly increased Rad50 level of 1.1±0.1, which was 1.3 times higher than in irradiated control cells (0.8±0.2).

Taken together, the Western blot analysis did not reveal any significant differences in the net expression of DNA-repair proteins hMre11, Rad51 and Rad50 between all tested groups. We therefore further analysed the subnuclear distribution of these proteins.

### hMre11, Rad51 and Rad50 nuclear focus formation

**Figure 4:**
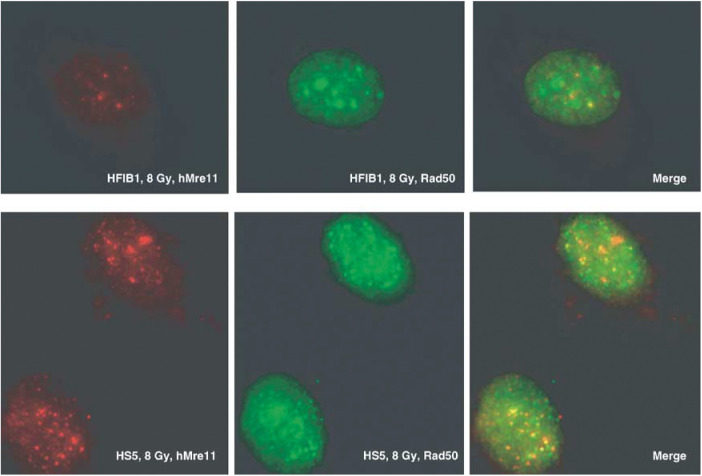
Immunofluorescence analysis of nuclear hMre11 and Rad50 foci in irradiated skin fibroblasts derived from a healthy individual (HFIB1, top) and from a hypersensitive cancer patient (HS5, bottom). Cells were irradiated with 8 Gy, fixed 2 h postirradiation and double stained with anti-hMre11 (red fluorescence) and anti-Rad50 (green fluorescence) antibodies. Right-hand images represent merged analysis.

The microphotographs in Figure 4

show the examples of nuclear foci that appeared in fibroblasts in response to IR, which were visualised by double immunofluorescence staining for hMre11 (left images) and Rad50 (middle images). The right-hand images are merged images. As seen from Figure 4, the brightly fluorescent foci are clearly distinguishable from the diffuse staining of the remaining nucleus. Such images were quantified by counting the number of foci-positive cells, and also by counting the number of foci per nucleus in the fraction of foci-positive cells.

**Figure 5:**
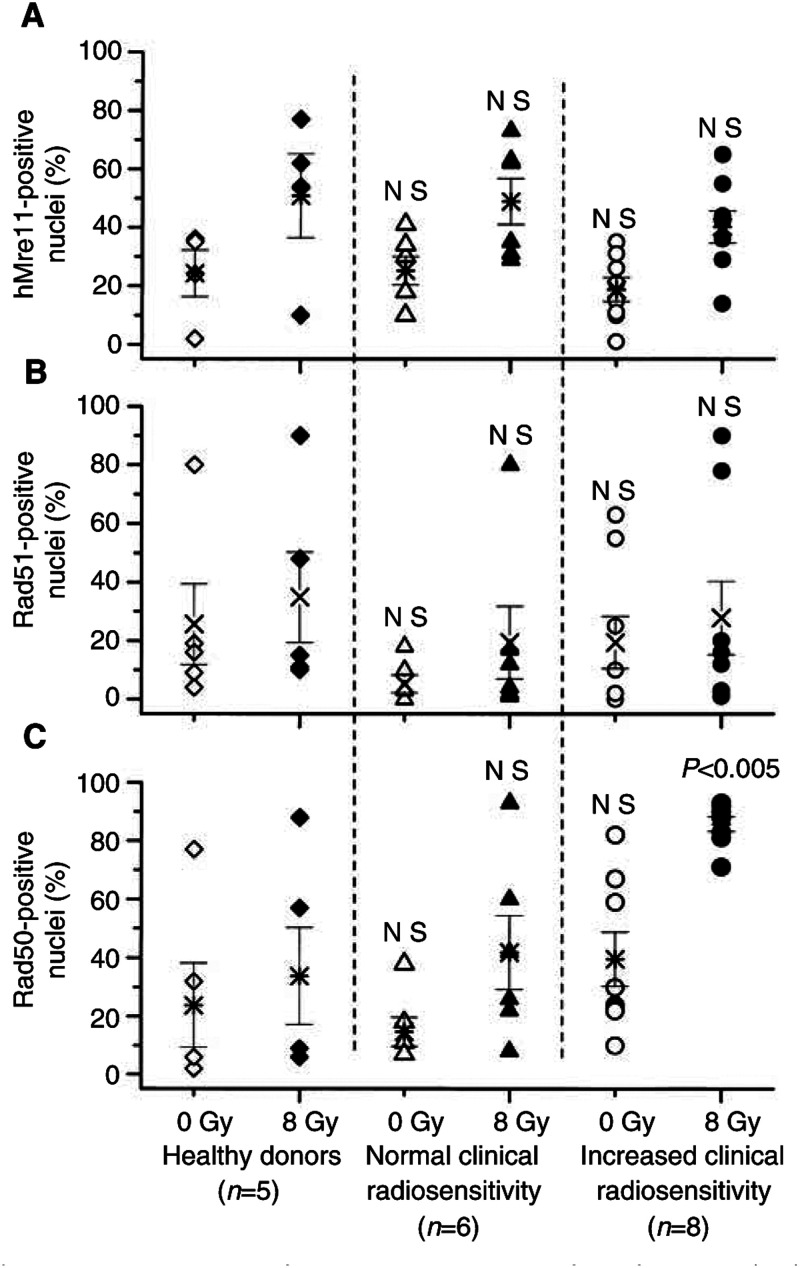
Percentages of cells containing nuclear foci of hMre11 (top), Rad51 (middle) and Rad50 (bottom) before (open symbols) and 2 h post-irradiation with 8 Gy (solid symbols). Each symbol (except stars) represents the mean value obtained for 100 cells derived from a given individual, in at least two independent experiments. Stars represent mean value (±s.e.) averaged through each tested group. ‘NS’ indicated that the difference was not highly significant (*P*>0.05).

For hMre11 (Figure 5A

) and Rad51 (Figure 5B), analysis of the fractions of foci-positive cells, as well as of the number of hMre11 and Rad51 foci per nucleus (data not shown) did not reveal any difference between all tested groups, either before or after irradiation. In contrast to the hMre11 and Rad51 data, the fraction of Rad50 foci-positive cells (Figure 5C, filled symbols) in the irradiated cells from hypersensitive cancer patients (88%) was significantly higher (<0.005) than in control cells (41%) and cells from cancer patients with normal reaction to RT (44%). Without irradiation (Figure 5C, open symbols), the fraction of Rad50 foci-positive nuclei in the hypersensitive cells (40%) was also apparently higher than in control (26%) or in cells from cancer patients with normal reaction to RT (15%). There might be several reasons for the wide scatter of the data points in Figure 5. Firstly, we used primary fibroblast strains, which are not monoclonal lines, but they are rather a heterogeneous population of cells in different stages of differentiation from fibroblasts to postmitotic fibrocytes (Bayreuther *et al*, 1988). Beside this, the cell lines were in different passage numbers ranging from 3 to 12. Secondly, the rather small group of patients and the heterogeneity of their clinical radiation responses may be further important reasons for the large data variability.

**Figure 6:**
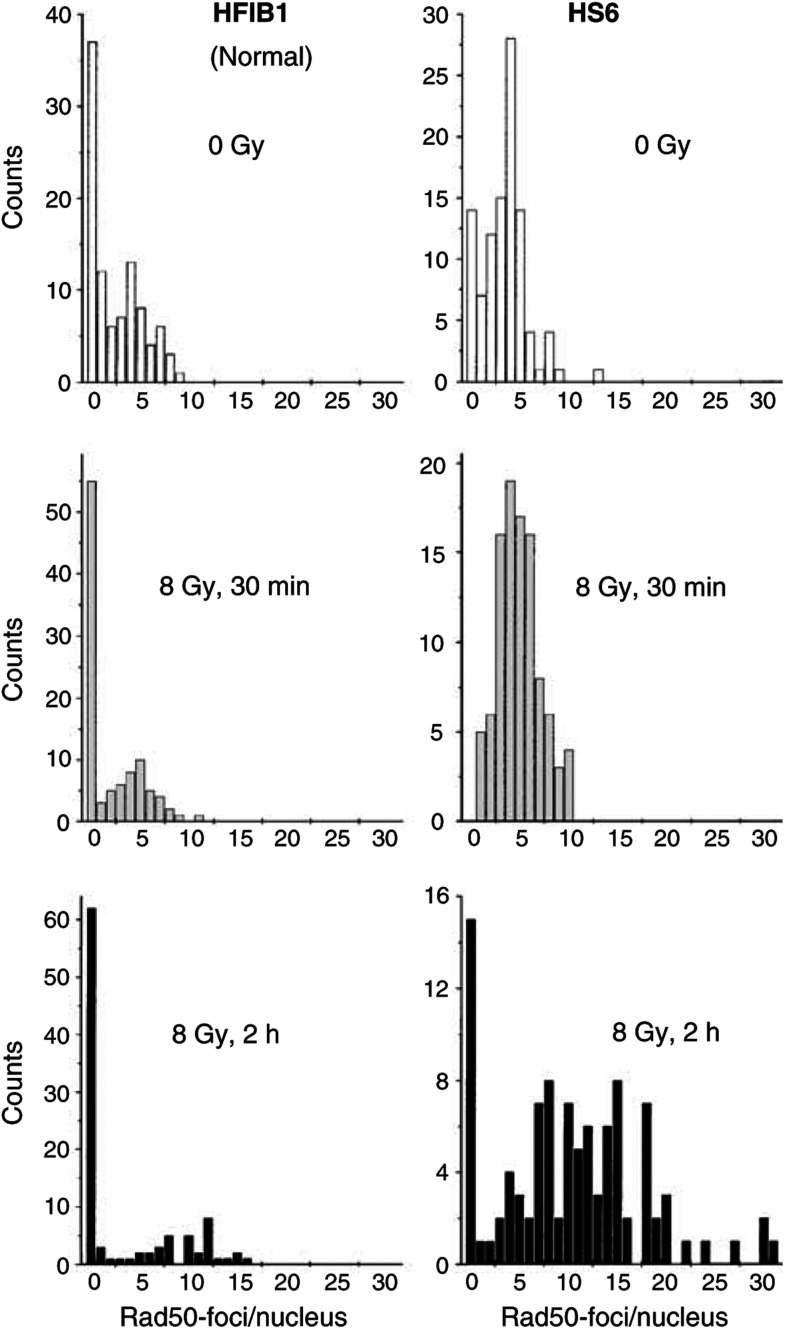
Histograms depicting the kinetics of Rad50 focus formation in normal cells (HFIB1, left column) and cells from a radiosensitive cancer patient (HS6). Cells were analysed for Rad50 focus induction before (top histograms), 30 min (middle) and 2 h (bottom histograms) after irradiation with 8 Gy. In total, 100 nuclei were counted per each time point.

In order to further characterise the observed increase of the fraction of Rad50 foci-positive nuclei in cells derived from hypersensitive cancer patients, we quantified the formation of Rad50 foci by counting their number per nucleus. Figure 6

illustrates typical distributions of Rad50 foci per nucleus in normal (HFIB1 strain, left-hand column) and hypersensitive cells (HS6 strain, right-hand column). The top, middle and bottom histograms in Figure 6 show the cells without irradition, as well as 30 min and 2 h after irradiation with 8 Gy, respectively. Without irradiation, the control HFIB1 strain derived from a healthy subject revealed a substantially larger fraction of Rad50 foci-negative cells (about 36%) than the hypersensitive HS6 cells (14%). At 30 min postirradiation, the foci-negative fraction in the hypersensitive HS6 strain disappeared, whereas the control strain (HFIB1) had a high fraction of foci-negative cells (about 55%). The mean numbers of foci per nucleus in the foci-positive fraction (about 5) were similar in both strains 30 min after irradiation. At 2 h after irradiation (Figure 6, bottom histograms), the Rad50 foci-negative fraction in the control strain remained nearly unchanged (about 60%), whereas the mean number of foci per nucleus (10) was twice as high as 30 min after irradiation. Compared to the data of normal cells obtained 2 h after irradiation, the hypersensitive cell line showed a substantially larger fraction of foci-positive cells (86 *vs* ∼40% in control) with a higher number of foci per nucleus (15 *vs* 10).

Analysis of the immunofluorescence data (Figures 4, 5 and 6) revealed that cells derived from hypersensitive cancer patients differed markedly in their Rad50 foci forming response to IR from the cells derived from healthy subjects and cells from cancer patients with normal clinical reaction to RT.

## DISCUSSION

Skin fibroblasts derived from the two groups of cancer patients were found to be more sensitive to X-irradiation *in vitro* than cells from apparently healthy donors when compared by the colony-forming assay several days or weeks after X-ray exposure (Figure 1 and Table 1). Thus, the mean SF2 value averaged through the hypersensitive group was significantly lower than in control. At the same time, the SF2 value for the group of hypersensitive cancer patients was very similar to that of the group of cancer patients with normal clinical reaction to RT. This means that in this sample the *in vitro* SF2 parameter did not discriminate between normal and increased acute clinical reactions during and after radiotherapy. Therefore, other cellular indicators were analysed for their correlation with the different responses of cancer patients to RT.

As already mentioned, DSBs are the most lethal form of DNA damage and they also represent the major group of DNA lesions induced by IR (for a review, see Jeggo, 1998). Therefore, genes involved in the DSBs processing and repair, such as hMre11, Rad50 and Rad51, might be promising molecular indicators of the radiation hypersensitivity *in vivo*. Particularly, identification of possible alterations in these genes or their proteins could be useful for predicting hypersensitivity to radiotherapy.

Despite the great variability of the cell lines used here with respect to the clinical radiosensitivity (i.e., grades 1–4 according to RTOG criteria), an extensive Western blot analysis did not reveal any differences in the expression and migration patterns of hMre11 and Rad50 proteins under *in vitro* conditions (Figures 2 and 3). Assuming that the detected *in vitro* levels of these proteins reflect those *in vivo*, this finding suggests that the net amounts of these DNA repair proteins do not account for the difference in radiation response *in vivo* observed during radiotherapy of these cancer patients. It should be noted, however, that, firstly, gene expression analysis based on the protein determination has obvious limitations mainly due to the constant level of expression during post-translational modifications. Secondly, due to its poor accuracy, the Western blot assay may be insufficiently sensitive to detect *subtle* differences between the cell lines derived from patients with different clinical reactions to RT. A poor sensitivity of Western blot has been pointed out by Carlomagno *et al* (2000), who demonstrated that the expression levels of nine different proteins, and among them Rad51, in 10 cell lines obtained from the skin biopsies of cancer patients with different clinical radiosensitivities were similar to those in three control cell lines. A recent study (Leong *et al*, 2003), in which cells from 36 patients were examined for defects in DNA ligase IV, XRCC4, Ku70 and Ku80 proteins using Western blot, also did not reveal any differences in the expression levels and migration patterns for the four proteins in all tested radiosensitive patients compared with controls.

In contrast to Carlomagno *et al* (2000), we observed in the present study a slight increase in the expression of Rad51 in skin fibroblasts derived from both groups of cancer patients compared with healthy group (Figure 3B).

The role of Rad51 protein in radiation sensitivity has been discussed controversially in the literature. Thus, overexpression of this protein has been found to correlate with increased cellular resistance against radiation (Vispé *et al*, 1998; Yanagisawa *et al*, 1998; Yanez and Porter, 1999). A 2–3-fold overexpression of Rad51 in CHO cells has stimulated the homologous recombination between integrated genes by 20-fold, indicating the key role of this protein in the intrachromosomal recombination pathway (Vispé *et al*, 1998). Increased concentrations of the Rad51 mRNA have also been shown by Northern blot in the radioresistant human KB carcinoma cell line N10 (Yanagisawa *et al*, 1998). Three human prostate cancer cell lines have exhibited increased radiosensitivity together with significant downregulation of Rad51, as compared to control cells (Collis *et al*, 2001). A 2–3-fold increase in homologous recombination and enhanced resistance to IR has been reported for cells overexpressing hRAD51 (Yanez and Porter, 1999).

On the contrary, overexpression of human Rad51 in CHO cells has been found to reduce the repair rate of DSBs by HR (Kim *et al*, 2001). These authors have also indicated that basal RAD51 gene expression correlates negatively with micronuclei induction in irradiated blood cells, and the level of Rad51 can partly explain the individual sensitivity to IR assessed by radiation-induced micronuclei. A 10-fold overexpression of Rad51 in HT1080 fibrosarcoma cell line has resulted in decreased plating efficiency and growth rate, in a dose-dependent manner with regard to the degree of overexpression (Flygare *et al*, 2001).

Taken together, the results of this study (Figures 2 and 3) and the controversial literature quoted above suggest that screening for abnormalities of DNA repair proteins, hMre11, Rad50 and Rad51, using Western blotting is unlikely to be useful for predicting clinical radiosensitivity. Therefore, we extended our experiments to account for the IR-induced nuclear foci formation of DNA repair proteins. Indeed, we found a protracted Rad50 foci formation in irradiated cells derived from cancer patients with increased early reactions to radiotherapy. Moreover, these cells displayed also an increased number of Rad50 foci per cell after irradiation. Our results are in agreement with an earlier report that the IR-induced foci response is increased for both hMre11 and Rad50 in a DSB repair-deficient 180BR cell line, which is also highly sensitive to IR (Maser *et al*, 1997).

It has been shown previously that IR-induced Rad50 nuclear focus formation in fibroblast cell lines is independent of the cell cycle (Yuan *et al*, 2003). Therefore, the alterations of the Rad50 foci formation in cells derived from hypersensitive cancer patients observed in the present study (Figures 4, 5C and 6) cannot be explained by the differences in the cell cycle distribution between the cell strains used. The observed increased number and altered distribution of Rad50 foci suggest a gene defect that impairs the ability to repair IR-induced DSBs in cells from hypersensitive cancer patients, thus decreasing the DNA restoration rate in these cells compared to cells derived from healthy subjects and cancer patients with normal reaction to RT.

## CONCLUSIONS

In this study, the colony-forming assay and Western blot analysis of the DNA repair proteins hMre11, Rad50 and Rad51 did not reveal any abnormalities in cellular radiosensitivity *in vitro* and in expression levels or migration patters of these proteins in the fibroblasts derived from the cancer patients with increased early reaction of normal tissue to radiotherapy. In contrast, the clinical radiation reaction might correlate with the impaired formation of the radiation-induced Rad50 foci that was assessed by immunofluorescence microscopy.
